# Pancreatic Neuroendocrine Tumor Leading to a Diagnosis of Multiple Endocrine Neoplasia Type 1

**DOI:** 10.1002/deo2.70160

**Published:** 2025-06-06

**Authors:** Noriyuki Hirakawa, Katsuya Kitamura, Kei Yamamoto, Kenichi Tadokoro, Yasunosuke Akita, Jun Uemura, Fumito Yamanishi, Masakazu Abe, Munehide Nakatsugawa, Takao Itoi

**Affiliations:** ^1^ Department of Gastroenterology and Hepatology Tokyo Medical University Hachioji Medical Center Tokyo Japan; ^2^ Department of Pathology Tokyo Medical University Hachioji Medical Center Tokyo Japan; ^3^ Department of Gastroenterology and Hepatology Tokyo Medical University Tokyo Japan

**Keywords:** endoscopic ultrasound | hyperparathyroidism | multiple endocrine neoplasia type 1 | pancreatic neuroendocrine tumor | pituitary adenoma

## Abstract

Pancreatic neuroendocrine neoplasms are rare but occasionally encountered. They are generally highly vascularized solid tumors, often round in shape with clear boundaries, defined contours, and a homogeneous internal structure. However, they can also present with atypical features, such as cystic degeneration, hemorrhage, calcification, and fibrosis, making diagnosis difficult in some cases. They are also known as comorbidities of multiple endocrine neoplasia type 1 (MEN1). This report describes a case in which endoscopic ultrasound (EUS) led to a diagnosis of MEN1. A 50‐year‐old man was referred to our hospital for examination of a mass in the pancreatic body. An EUS‐guided fine‐needle biopsy was performed, and a histological diagnosis of neuroendocrine tumor (NET) was made. In addition, the NET was also identified in the duodenum. Serum calcium and parathyroid hormone levels were elevated. Examination of the parathyroid and pituitary glands revealed concurrent hyperparathyroidism and a pituitary adenoma, confirming the diagnosis of MEN1, including a NET in the duodenum.

## 1 Introduction

Pancreatic neuroendocrine neoplasms (P‐NENs) are relatively rare, with an estimated incidence of 1.27 per 100,000 population [1, 2]. It has been reported that 4.3%–10% of P‐NENs are derived from multiple endocrine neoplasia type 1 (MEN1) [1, 2]. MEN1 is an autosomal dominant disease that causes hyperplasia, adenomas, and tumors in various endocrine organs. Inactivating mutation of the tumor suppressor gene *MEN1* leads to the development of functional and non‐functional tumors in various organs. Hyperparathyroidism, gastroenteropancreatic NENs, and pituitary adenomas are characteristic manifestations, and a definitive diagnosis is made if there are two or more findings. The penetrance rates of hyperparathyroidism, gastroenteropancreatic NENs, and pituitary adenomas are reported to be 94.4%, 58.6%, and 49.6%, respectively [3, 4]. This report describes a case in which a pancreatic tumor detected on computed tomography (CT) was identified as a neuroendocrine tumor (NET) by endoscopic ultrasound (EUS), and tests based on clinical symptoms led to a diagnosis of MEN1.

## 2 Case Report

The patient was a 50‐year‐old man with a history of alcohol‐induced chronic pancreatitis, and perforated duodenal ulcer. A mass in the pancreatic body was found on a CT at the previous hospital, and he was referred to our hospital. The patient did not report any subjective symptoms. His laboratory and basal endocrine findings are shown in Table 1. His corrected serum calcium level was 11.1 mg/dL, his serum intact parathyroid hormone level was 243.7 pg/mL (normal range, 10–65), and his serum gastrin was 280 pg/mL (normal range, 37–172).

**TABLE 1:** Laboratory and basal endocrinological findings.

Parameter	Values	Reference range
Serum albumin, g/Dl	3.7	3.8–5.3
Serum calcium, mg/dL	11.1	8.4–10.2
Serum amylase, U/L	78	44–132
Serum phosphorus, mg/dL	2.5	2.5–4.5
Alkaline phosphatase, U/L	115	104–338
CEA, ng/mL	<1.7	<5
CA19‐9, ng/mL	6.42	<37
HbA1c, %	6.1	4.3–5.8
Fasting serum glucose, mg/dL	109	10–110
Insulin, µU/mL	2	2–12
HOMA‐R	0.5	1.6–2.5
Intact PTH, pg/mL	243.7	10–65
ACTH, pg/mL	39.8	7–56
Cortisol,µg/mL	14.52	4.0–19.3
TSH, µU/mL	0.60	0.49–4.67
Free T3, pg/mL	3.70	1.45–3.48
Free T4, pg/mL	1.01	0.71–1.85
Prolactin, ng/mL	178	6.12–30.54
Calcitonin, pg/mL	1.22	<5.15
LH, mIU/mL	11.62	5.72–64.31
FSH, mIU/mL	19.84	<157.79
Growth hormone, ng/mL	0.45	0.010–3.607
IGF‐1, ng/mL	120	79–215
Gastrin, pg/mL	230	37–172
Glucagon, pg/mL	22.5	23–197

Abbreviations: ACTH: adrenocorticotropic hormone, CA19‐9: carbohydrate antigen19‐9, CEA: carcinoembryonic antigen, FSH: follicle stimulating hormone, HOMA‐R: homeostasis model assessment‐resistance, IGF‐1: insulin‐like growth factor, LH: luteinizing hormone, PTH: parathyroid hormone, T3: triiodothyronine, T4: thyroxine, TSH: thyroid stimulating hormone.

**FIGURE 1:**
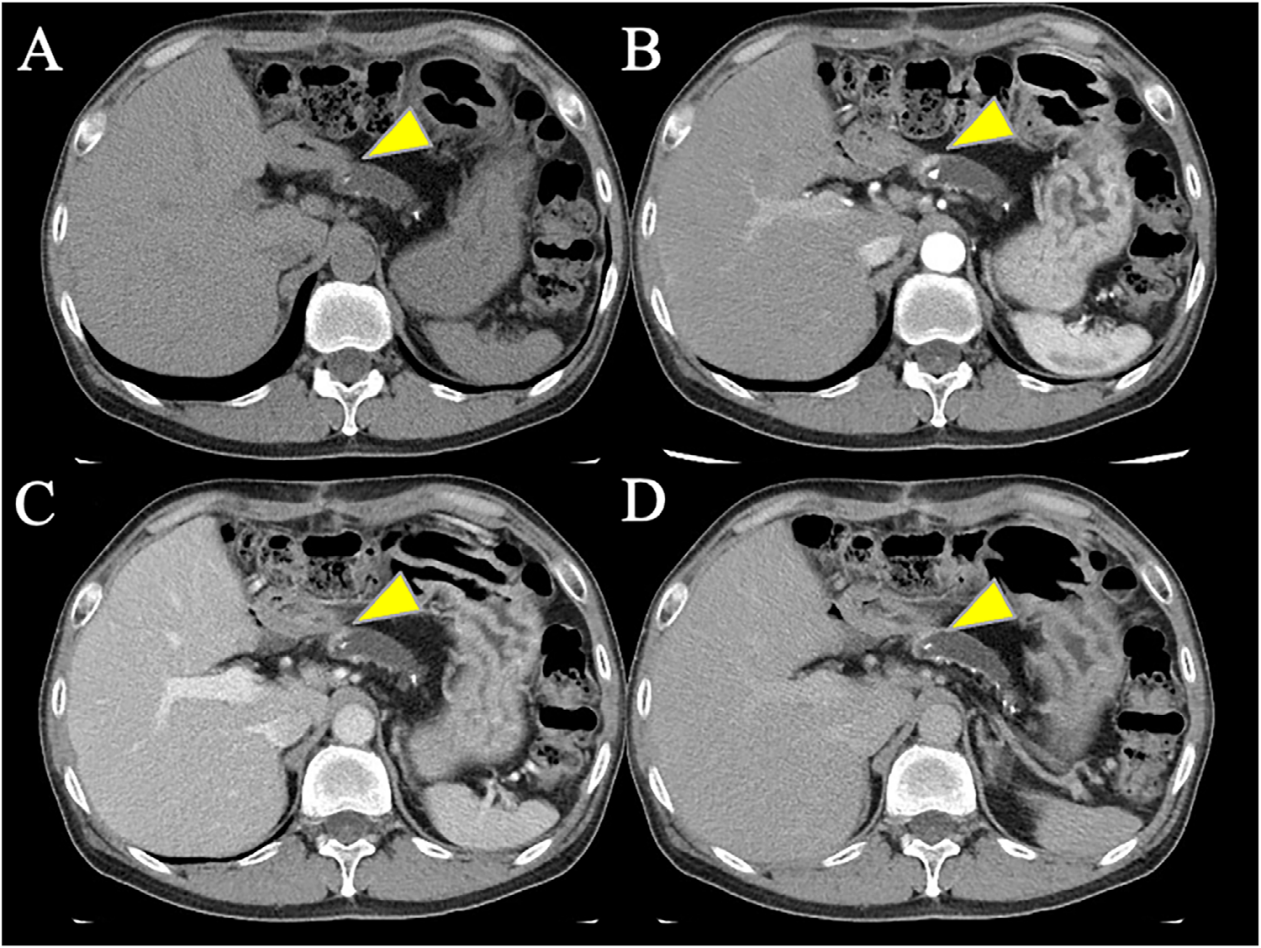
Findings on computed tomography (CT). (A) Plain. (B) Arterial phase. (C) Portal venous phase. (D) Delayed phase. On contrast‐enhanced CT, a slight hypervascular mass was observed in the arterial phase at the location indicated by the arrow.

An abdominal CT scan revealed overall atrophy, calcification, and pancreatic stones in the pancreatic parenchyma due to chronic pancreatitis, with dilation of the pancreatic duct from the body to the tail. An abdominal contrast‐enhanced CT showed a slight hypervascular mass in the pancreatic body (Figure 1) and a hypervascular mass in the tail (Figure S1A). Magnetic resonance imaging (MRI) showed stones within the pancreatic duct and dilation of the pancreatic duct (Figure S2). The site of the stricture was isointense, and no diffusion restriction was observed on diffusion‐weighted images (b = 800). Somatostatin receptor scintigraphy revealed abnormal accumulation in the pancreatic body (Figure S3).

**FIGURE 2:**
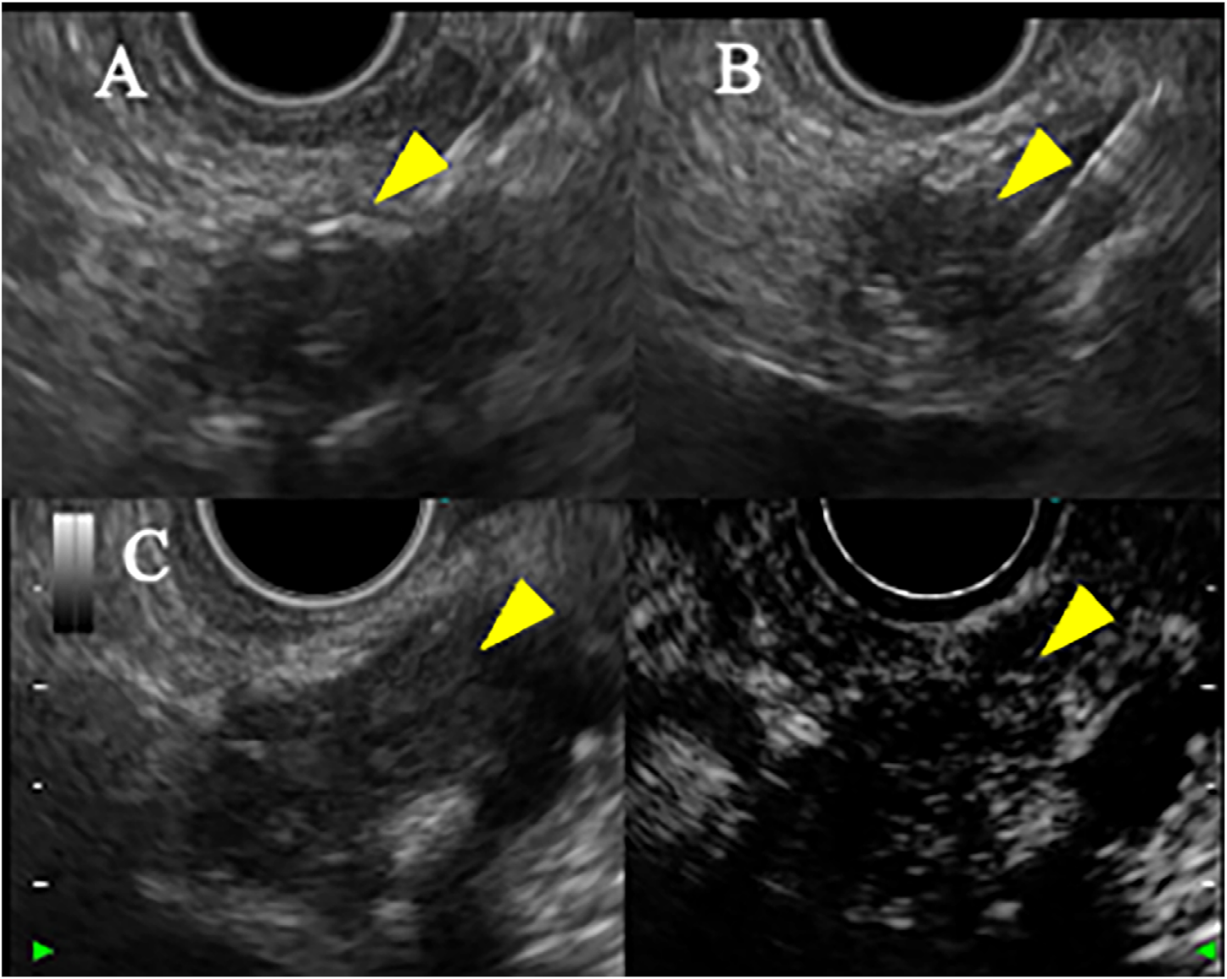
Findings on endoscopic ultrasound. (A) A 20‐mm hypoechoic mass with relatively clear borders was seen in the pancreatic body. (B) Endoscopic ultrasound‐guided fine‐needle biopsy was performed on the mass in the pancreatic body via the stomach. (C) Contrast‐enhanced harmonic endoscopic ultrasound showed a slight enhancement of the mass in the pancreatic body from an early stage.

**FIGURE 3:**
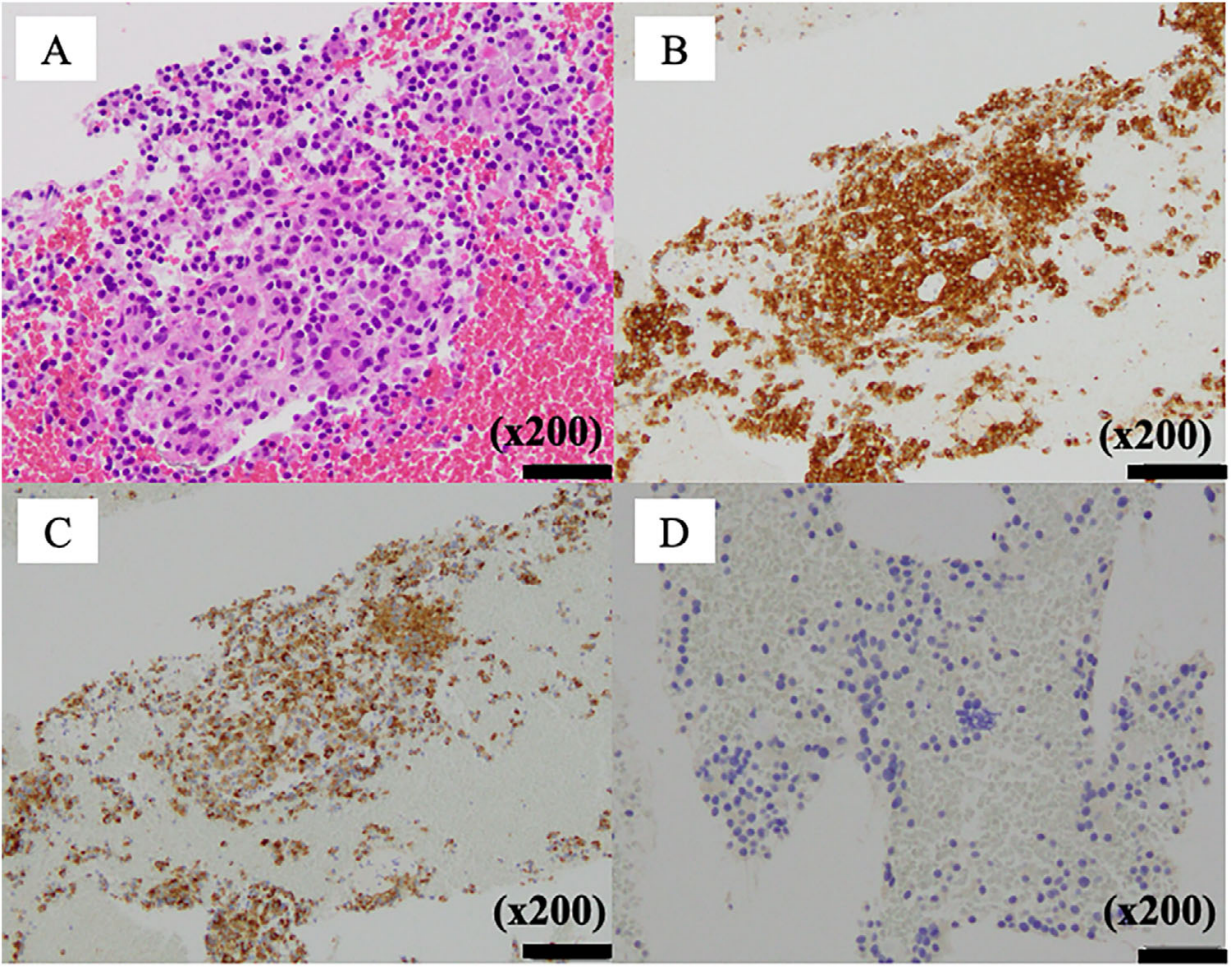
Microscopic observations for the pancreatic tumor. A pancreatic biopsy was performed, and the sample was analyzed by hematoxylin‐eosin staining (A) and immunohistochemical detection using (B) anti‐synaptophysin, (C) anti‐chromogranin A, and (D) anti‐gastrin. The tumor cells had uniform rounded nuclei and pale acidophilic cytoplasm. Scale bar, 50 µm. No mitotic cells were found. The tumor showed synaptophysin and chromogranin positivity, with a Ki‐67 labeling index of less than 1%. The tumor did not express gastrin.

EUS showed a 20‐mm hypoechoic mass in the pancreatic body with relatively clear borders, well‐defined margins, a homogeneous interior (Figure 2), and a similar hypoechoic mass measuring 8 mm in the pancreatic tail (Figure S1B). Contrast‐enhanced harmonic EUS with a sonographic contrast agent (Sonazoid; Daiichi‐Sankyo, Tokyo, Japan) showed enhancement of the mass in the pancreatic body from an early stage. EUS‐guided fine‐needle biopsy (FNB) with a 22‐gauge needle (Acquire; Boston Scientific, Marlborough, MA, USA) was performed on the mass in the pancreatic body via the stomach. Pathology showed tumor cells with uniform rounded nuclei and pale acidophilic cytoplasm. The diagnosis was pancreatic NET (P‐NET) (G1) (Figure 3). Immunostaining showed negativity for gastrin, insulin, and glucagon, leading to a diagnosis of non‐functional NET.

Esophagogastroduodenoscopy (EGD) was performed as the screening for gastrointestinal NET and showed a raised lesion in the descending portion of the duodenum (Figure S4). Boring biopsy revealed a lesion characteristic of NET. The tumor showed gastrin positivity and was diagnosed as gastrinoma (Figure S5).

No hereditary disorders were detected in his blood relatives. However, blood tests showed elevated serum calcium and parathyroid hormone levels, leading to suspicion of MEN1. A cervical contrast‐enhanced CT and ultrasound examination showed a 10‐mm nodule with calcifications on the posterior aspect of the right lobe of the thyroid gland (Figure S6). MRI of the head revealed a tumor with a multilocular cyst on the left side of the pituitary gland (Figure S7).

He underwent genetic testing after giving informed consent and receiving genetic counseling. The diagnosis of MEN1 was subsequently made at another medical facility by hereditary tumor tests.

A bilateral parathyroidectomy was performed for the parathyroid adenoma and hyperparathyroidism. The tumor showed parathyroid hormone positivity and the diagnosis was confirmed to be parathyroid adenoma (Figure S8). The pituitary adenoma is asymptomatic and is being followed up. The P‐NET was 20 mm, and considering the patient's history of duodenal ulcer and gastrinoma, surgical intervention is being considered for the future.

## 3 Discussion

P‐NENs may be functional (e.g., insulinoma or gastrinoma) or non‐functional, with one study in Japan finding that 65.5% of P‐NENs were non‐functional and 20.9% were functional [1]. Compared with functional P‐NENs, non‐functional P‐NENs tend to be larger at the time of detection because of their lack of symptoms and are generally considered to have a poorer prognosis. It is estimated that 10%–20% of P‐NENs are associated with hereditary diseases, the most representative of which are MEN1, von Hippel‐Lindau disease, tuberous sclerosis complex, and neurofibromatosis. Furthermore, 4.3%–10% of P‐NENs are reported to be derived from MEN1 [1, 2].

This case was referred for evaluation of a mass in the pancreatic body. Unlike a typical P‐NEN, the pancreatic mass showed relatively poor enhancement on contrast‐enhanced CT. It is known that the poorer the contrast enhancement of a P‐NEN, the higher the likelihood of malignancy [5]. It is not uncommon for tumors to develop fibrosis within the stromal tissue, and one study found stromal fibrosis within the tumor in 82% of P‐NENs [6]. However, when the tumor contains a significant amount of fibrous stroma, it may not enhance early and show a hypoenhanced or gradually enhancing pattern, which can complicate the differentiation from pancreatic cancer [7]. In addition, increased proinflammatory changes with chronic pancreatitis may hypothetically contribute to carcinogenesis in islet cells [8]. In this case, the poor contrast enhancement was interpreted as fibrosis resulting from inflammation caused by chronic pancreatitis affecting the tumor. Furthermore, a stricture of the pancreatic duct and dilatation of the distal pancreatic duct were observed. PNETs with pancreatic duct involvement have been reported to be more fibrotic in adjacent structures [9], and in this case, fibrosis was also observed in the pathological specimen, suggesting that the tumor had affected the pancreatic duct.

The imaging capability of EUS is superior to that of contrast‐enhanced CT for P‐NENs, and contrast‐enhanced harmonic EUS is now widely used for the diagnosis and differentiation of pancreaticobiliary diseases. Furthermore, EUS‐guided FNB can facilitate both the pathological diagnosis and grading of malignancy. EUS has also been reported to contribute to improved rates of diagnosis of lesions in the adrenal cortex, which are considered to be the fourth most common in MEN1 [10].

In this case, for pathological diagnosis, EUS‐FNB was performed for the P‐NET, and a boring biopsy was performed for the duodenal NET. This case was rare, presenting with not only the P‐NET, the parathyroid adenoma, and the pituitary adenoma but also the duodenal NET. Among MEN1‐associated P‐NENs, gastrinomas (40%), insulinomas (10%), and non‐functional tumors (20%) are common. While gastrinomas frequently develop in the duodenum and pancreas, this case involved a functional gastrinoma in the duodenum and a non‐functional tumor in the pancreas. Tumor formation because of MEN1 is believed to result from inactivating mutations in the tumor suppressor gene *MEN1*. The different pathological results in each organ, such as adenoma in the pituitary and NET in the gastrointestinal tract, are thought to result from a lack of correlation between the phenotypes of gene mutations and the specific organs.

NENs associated with MEN1 tend to be multiple. Regardless of the presence or absence of MEN1, functional tumors are an indication for treatment. For nonfunctional pancreatic NETs in MEN1 cases, surgical resection is recommended when the tumor size exceeds 2 cm, in view of the risk of liver metastasis. In this case, resection was considered for the functional NET in the duodenum and the nonfunctional NET in the pancreas. Distal pancreatectomy combined with duodenal resection, or total pancreatectomy, was planned.

MEN1 differs from non‐hereditary tumors in certain aspects of its clinical presentation, which can lead to different treatment strategies. The treatment for P‐NENs also varies depending on whether MEN1 is present, so it is essential to always consider MEN1 during the diagnosis of P‐NENs and to check for associated conditions. Furthermore, given the high incidence of P‐NENs in patients with MEN1, it may be worthwhile considering prophylactic EUS. In clinical practice, it is important to narrow down cases that are suspicious for MEN1 and confirm the diagnosis.

## Conflicts of Interest

Takao Itoi is the Editor‐in‐Chief of DEN Open. Takao Itoi is a consultant for Olympus.

## Supporting information




**FIGURE S1** Findings on imaging of the mass in the pancreatic tail. (A) On computed tomography. A hypervascular mass was observed in the arterial phase at the location indicated by the arrow. (B) On endoscopic ultrasound, an 8‐mm hypoechoic mass with relatively clear borders was seen in the pancreatic tail.
**FIGURE S2** Findings on magnetic resonance imaging. (A) T1‐weighted images. (B) T2‐ weighted images. (C) Diffusion‐weighted images. (D) Magnetic resonance cholangiopancreatography. The magnetic resonance images did not show any obvious tumors, and there was no diffusion restriction on the diffusion‐weighted images.
**FIGURE S3** Findings on somatostatin receptor scintigraphy. Abnormal accumulation is indicated by the arrows (A) and (B) in the body of the pancreas.
**FIGURE S4** Findings on esophagogastroduodenoscopy. (A) A raised lesion was seen in the descending portion of the duodenum. The central portion of the tumor showed mild depression. (B) Obvious epithelial changes were not observed.
**FIGURE S5** A duodenal biopsy was performed, and the sample was analyzed by hematoxylin‐eosin staining (A) and immunohistochemical detection using (B) anti‐synaptophysin, (C) anti‐chromogranin A, and (D) anti‐gastrin. Scale bar, 50 µm. The tumor cells had uniform rounded nuclei, formed a vesicular shape, and showed an infiltrative growth pattern. The tumor showed synaptophysin, chromogranin A, with a Ki‐67 labeling index of 1%, and Gastrin positivity and was diagnosed as gastrinoma.
**FIGURE S6** Findings on imaging of the thyroid gland. (A) Contrast‐enhanced computed tomography of the neck showed a 10‐mm nodule with calcifications on the posterior aspect of the right lobe. (B) Ultrasound examination showed an irregular hypoechoic nodule with internal heterogeneity.
**FIGURE S7** Findings on magnetic resonance imaging of the pituitary gland. (A) T1‐weighted images. (B) T2‐weighted images. Imaging of the head revealed a tumor with a multilocular cyst on the left side of the pituitary gland, with high signal intensity within the cyst on T2‐weighted images and low signal intensity on T1‐weighted images.
**FIGURE S8** Microscopic observations for the parathyroid tumor. The parathyroid tumor was surgically resected, and the sample was analyzed by (A) hematoxylin‐eosin staining and (B) immunohistochemical detection using anti‐parathyroid hormone. The tumor cells had uniform rounded nuclei and grew in a cordate fashion with capillaries. The tumor showed parathyroid hormone positivity and was diagnosed as parathyroid adenoma. Scale bar, 50 µm.
