# Supplementary figures and images for: Pancreatic Neuroendocrine Tumor Leading to a Diagnosis of Multiple Endocrine Neoplasia Type 1
Source: DEN Open. 2025 Jun 6;6(1):e70160. doi: 10.1002/deo2.70160 (PMC12143420; doi:10.1002/deo2.70160)

Figure S1.


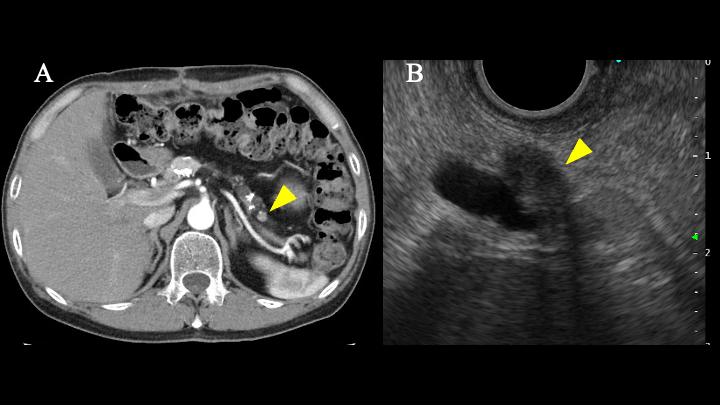


Figure S2.


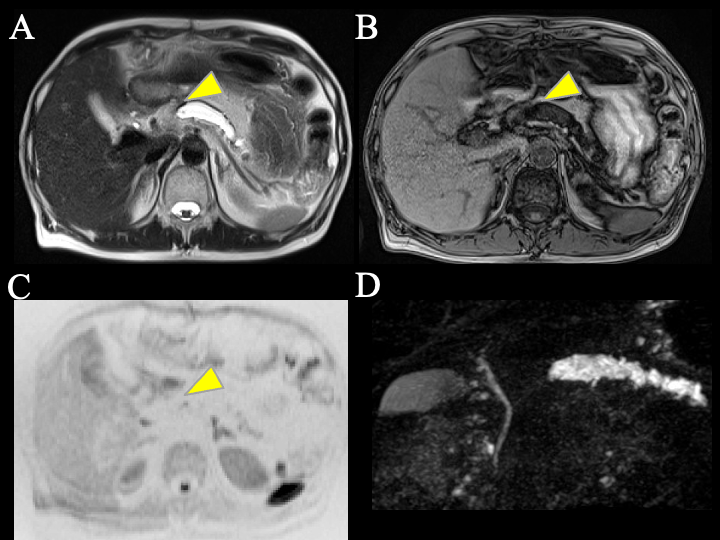


Figure S3.


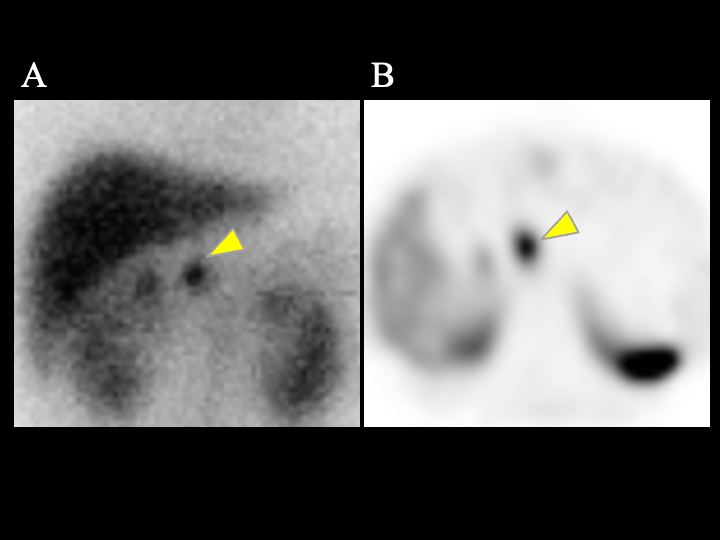


Figure S4.


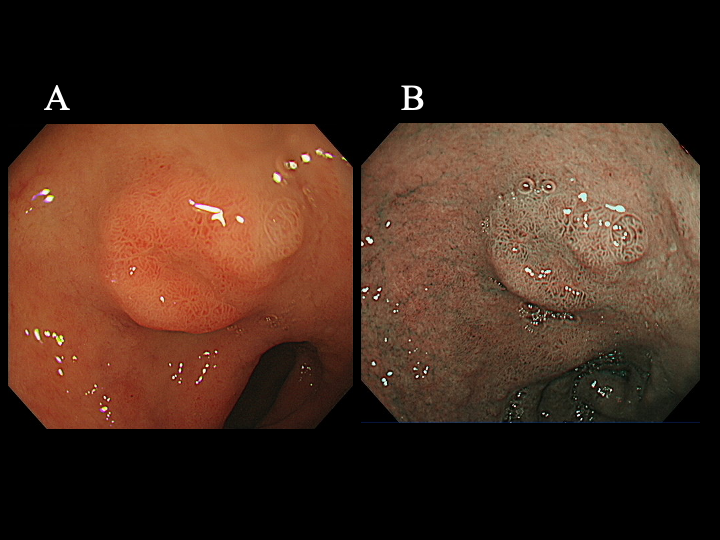


Figure S5.


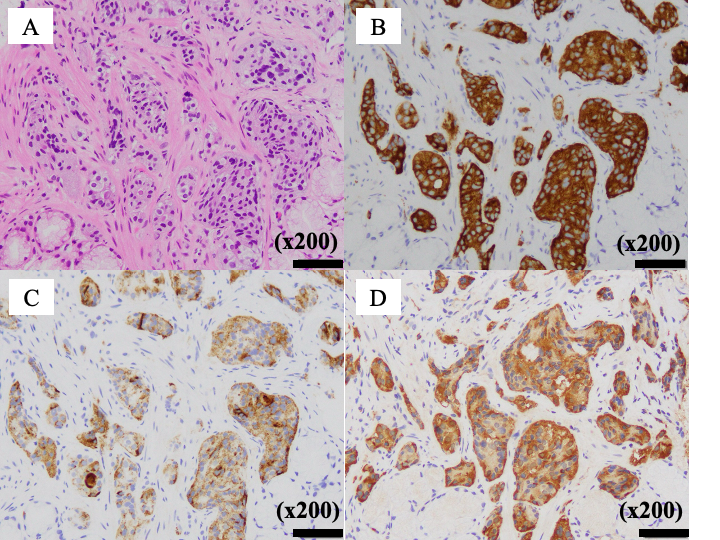


Figure S6.


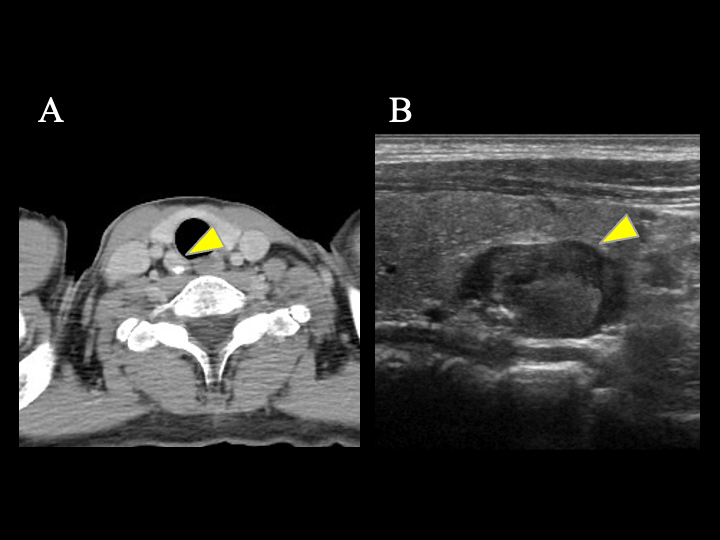


Figure S7.


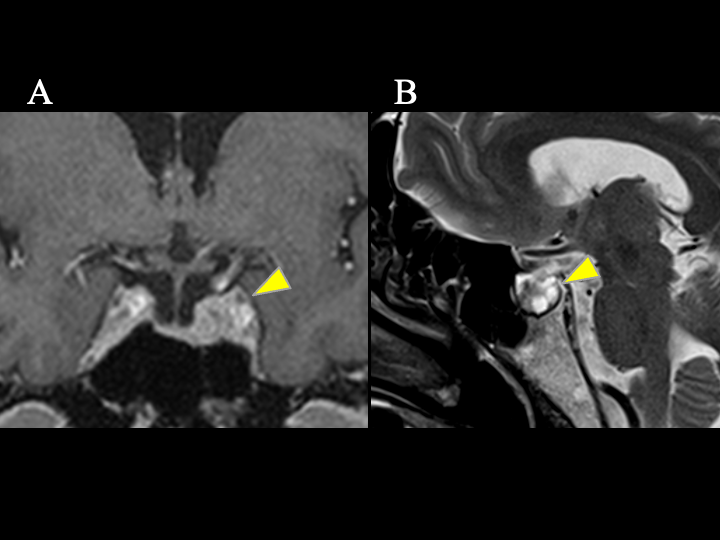


Figure S8.


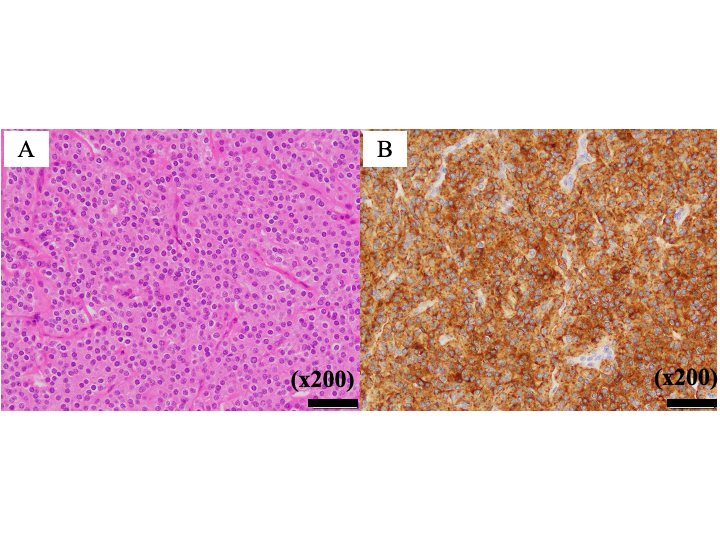

Supplement: Supplementary file 1 — FIGURE S1 Findings on imaging of the mass in the pancreatic tail. (A) On computed tomography. A hypervascular mass was observed in the arterial phase at the location indicated by the arrow. (B) On endoscopic ultrasound, an 8‐mm hypoechoic mass with relatively clear borders was seen in the pancreatic tail. FIGURE S2 Findings on magnetic resonance imaging. (A) T1‐weighted images. (B) T2‐ weighted images. (C) Diffusion‐weighted images. (D) Magnetic resonance cholangiopancreatography. The magnetic resonance images did not show any obvious tumors, and there was no diffusion restriction on the diffusion‐weighted images. FIGURE S3 Findings on somatostatin receptor scintigraphy. Abnormal accumulation is indicated by the arrows (A) and (B) in the body of the pancreas. FIGURE S4 Findings on esophagogastroduodenoscopy. (A) A raised lesion was seen in the descending portion of the duodenum. The central portion of the tumor showed mild depression. (B) Obvious epithelial changes were not observed. FIGURE S5 A duodenal biopsy was performed, and the sample was analyzed by hematoxylin‐eosin staining (A) and immunohistochemical detection using (B) anti‐synaptophysin, (C) anti‐chromogranin A, and (D) anti‐gastrin. Scale bar, 50 µm. The tumor cells had uniform rounded nuclei, formed a vesicular shape, and showed an infiltrative growth pattern. The tumor showed synaptophysin, chromogranin A, with a Ki‐67 labeling index of 1%, and Gastrin positivity and was diagnosed as gastrinoma. FIGURE S6 Findings on imaging of the thyroid gland. (A) Contrast‐enhanced computed tomography of the neck showed a 10‐mm nodule with calcifications on the posterior aspect of the right lobe. (B) Ultrasound examination showed an irregular hypoechoic nodule with internal heterogeneity. FIGURE S7 Findings on magnetic resonance imaging of the pituitary gland. (A) T1‐weighted images. (B) T2‐weighted images. Imaging of the head revealed a tumor with a multilocular cyst on the left side of the pituitary gland, with h [file DEO2-6-e70160-s001.docx]
